# Arterial thrombotic complications in COVID-19 patients

**DOI:** 10.1016/j.jvscit.2020.06.012

**Published:** 2020-07-17

**Authors:** Stef Levolger, Reinoud P.H. Bokkers, Jan Wille, Rogier H.J. Kropman, Jean-Paul P.M. de Vries

**Affiliations:** a Department of Radiology, Medical Imaging Center, University Medical Center Groningen, University of Groningen, Groningen, The Netherlands; b Department of Vascular Surgery, St. Antonius Hospital, Nieuwegein, The Netherlands; c Division of Vascular Surgery, Department of Surgery, University Medical Center Groningen, University of Groningen, Groningen, The Netherlands

**Keywords:** Arterial occlusion, Thrombosis, Ischemia, COVID-19, SARS-CoV-2

## Abstract

The coronavirus disease 2019 (COVID-19), a viral respiratory illness caused by the severe acute respiratory syndrome coronavirus 2 (SARS-CoV-2), has been described to predispose to thrombotic disease in both the venous and arterial circulations. We report four cases of an acute arterial occlusion in COVID-19 patients and literature review on the occurrence of arterial thrombosis in patients with COVID-19. Our findings demonstrate that physicians should be vigilant for signs of thrombotic complications in both hospitalized and new COVID-19 patients.

## Case report

We report four cases of acute arterial occlusive disease or ischemia in patients with the coronavirus disease 2019 (COVID-19) that is caused by the virus designated severe acute respiratory syndrome coronavirus 2 (SARS-CoV-2). The patients presented at two Dutch hospitals (one university hospital and one large teaching hospital). Since the first presentation of a COVID-19 patient in The Netherlands on February 27 to May 1, there have been 378 reverse transcriptase-polymerase chain reaction-confirmed patients admitted to our two hospitals. Four cases involved an acute arterial occlusion. Medical history, smoking status, laboratory results, and anticoagulation therapy are detailed in Table I.

**Table I:** Identified cases of acute arterial occlusive disease or ischemia in patients with COVID-19

	Case 1	Case 2	Case 3	Case 4
Medical history	None	Diabetes mellitus	Gout	Obstructive sleep apnea, obesity
Smoking	Nonsmoker	Nonsmoker	Smoking cessation 3 years prior	Nonsmoker
Laboratory results				
CRP, mg/L	234	100	4.7	339
Leukocytes, 10^9^/L	8.2	13	7.2	23.6
Thrombocytes, 10^9^/L	262	458	185	237
LDH, U/L	868	294	186	421
aPTT, seconds	N/A	34	35	34
PT, seconds	N/A	14	N/A	14.7
INR	N/A	N/A	1.1	N/A
D-dimer, μg/L	N/A	N/A	N/A	28,186
Anticoagulation therapy				
At admission	–	–	–	–
Post-therapy	Apixaban, 5 mg twice daily	Heparin IV	Clopidogrel, 75 mg once daily Nadroparin (Fraxiparine), 2850 IU once daily	Heparin IV^a^
At discharge	Apixaban, 5 mg twice daily	Rivaroxaban, 10 mg once daily	–	Acenocoumarol^a^

*aPTT,* Activated partial thromboplastin time; *CRP,* C-reactive protein; *INR,* international normalized ratio; *IV,* intravenous; *LDH,* lactate dehydrogenase; *N/A,* not available; *PT,* prothrombin time.

a In case 4, the patient was treated with heparin intravenously after therapy because of concomitant acute kidney failure, for which continuous venovenous hemofiltration was indicated. Acute atrial fibrillation and subsegmental pulmonary embolisms developed during recovery, for which acenocoumarol was started before discharge to a referral hospital.

**Fig 1:**
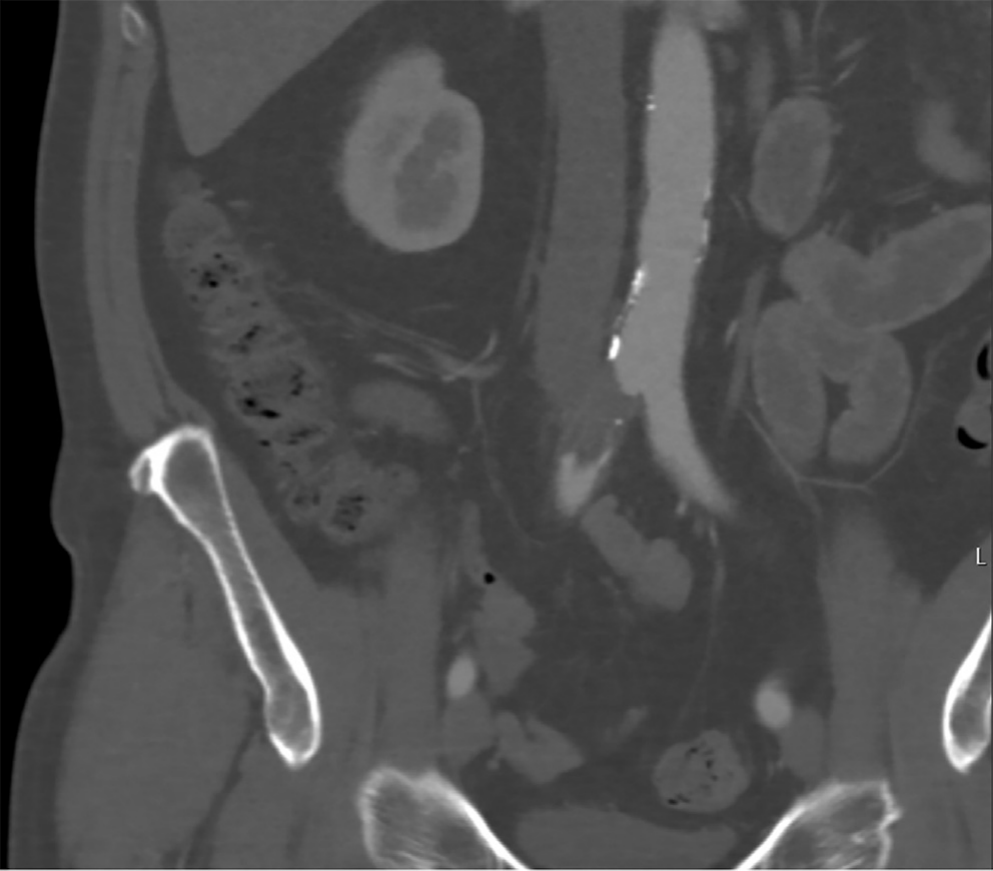
A 50-year-old COVID-19-positive man with no medical history developed acute claudication of the right limb without neurologic deficits 3 days after admission. Computed tomography angiography (CTA) showed short occlusion of the right common iliac artery.

The first patient, a 50-year-old healthy man, was admitted because of pneumonia, for which he received supplemental oxygen and chloroquine. Three days after admission, the patient developed acute claudication of the right limb without neurologic deficits. Computed tomography angiography (CTA) showed a short occlusion (3.5 cm) of the right common iliac artery (Fig 1). Surgical embolectomy was not possible because of a high risk for general anesthesia in relation to COVID-19, and thrombolytic therapy was not available because of capacity issues. After 3 days of systemic therapeutic heparin treatment, the patient was discharged home with mild claudication. However, 20 days later, the patient was readmitted with acute ischemia of both legs. CTA showed persistent occlusion of the right common iliac artery. A new thrombus was present at the left tibial-fibular trunk (TTF). The patient subsequently received alteplase for the right common iliac artery and left TTF. Owing to the dislodgment of thrombus to the right TTF and persistent ischemia, infragenual exploration and open thrombectomy were performed, with good clinical outcome.

**Fig 2:**
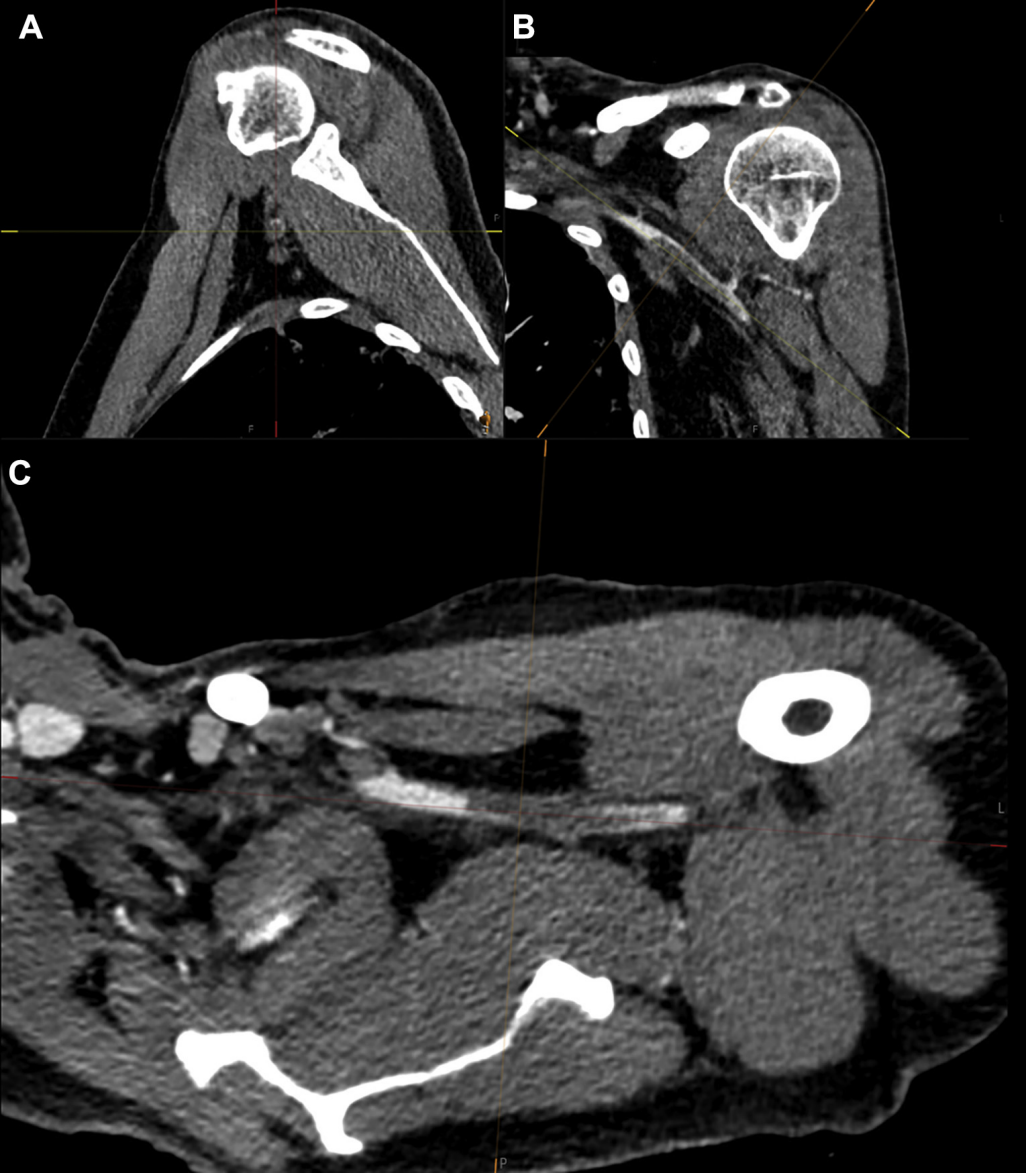
A 55-year-old COVID-19-positive man with a medical history of diabetes with oral metformin use presented with a pale, pulseless left hand without muscle weakness and minimal sensory loss of the fingers. Acquired computed tomography angiography (CTA) imaging showed subclavian artery occlusion. **A,** Sagittal view. **B,** Coronal view. **C,** Axial view.

The second patient, a nonsmoking 55-year-old man, was referred with a pale pulseless left hand. There was no muscle weakness, with minimal sensory loss of the fingers. CTA was performed and showed a subclavian artery occlusion (Fig 2). One week before, the patient had had a fever without other symptoms. At presentation, the patient had no pulmonary symptoms, fever, or dyspnea. The saturation was 95%. The patient was treated with therapeutic heparin systemically. Because of the absence of fever and hypoxia, no treatment was started for the COVID-19 infection. After 1 day of heparin treatment, distal pulses were still absent, but refill was normalized, and the function of the hand was completely normalized, with no sensory loss. After 2 days, rivaroxaban was started, and the patient was discharged home.

**Fig 3:**
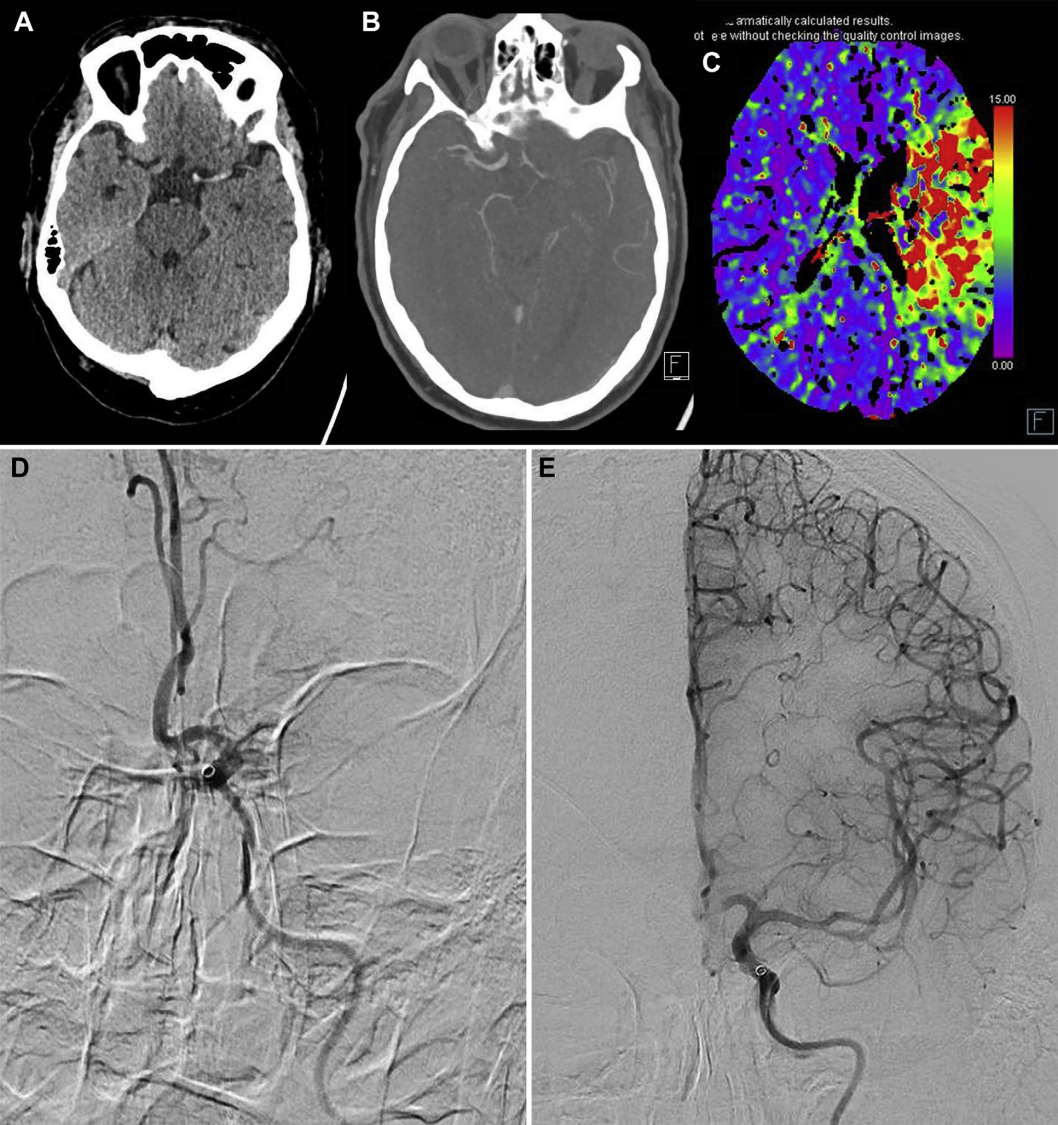
A 62-year-old COVID-19-positive man presented with right-sided hemiparesis. **A-C,** Cerebral computed tomography showed **(A)** a left dense media sign, **(B)** occlusion on computed tomography angiography (CTA), and **(C)** corresponding perfusion defect. **D,** Digital subtraction angiography confirmed an M1 occlusion. **E,** After intra-arterial thrombectomy, cerebral blood flow was restored (Thrombolysis in Cerebral Infarction grade 2C).

The third patient, a 62-year-old man, presented with right-sided hemiparesis. Computed tomography imaging showed dense media with a corresponding perfusion defect as well as M1 occlusion on CTA with subtotal stenosis of the internal carotid artery origin (Fig 3). There was no known history of internal carotid artery stenosis, and CTA showed minimal calcified atherosclerosis. The patient underwent intra-arterial thrombectomy with nearly complete reperfusion, except for some distal cortical emboli. On the second day of admission, the patient developed fever and cough due to COVID-19. Laboratory results were normal. Further symptoms were mild, without the need for supplemental oxygen or other treatment for COVID-19. As of June 9, 2020, the patient is still admitted for neurologic rehabilitation.

The fourth patient, a 58-year-old man, who presented with dyspnea and abdominal pain that had gradually developed within the past 2 weeks. The patient was admitted to the intensive care unit (ICU) for respiratory distress the same day. Gastric retention and abdominal distention were noted during the admission; abdominal portal-venous computed tomography imaging was performed, showing dilated small bowel loops, signs of bowel wall ischemia, an adjacent fluid collection, and splenic and renal infarctions without macrovascular arterial occlusion. Nonsignificant stenosing soft plaque was present in the proximal superior mesenteric artery. Subsequent laparotomy was performed, and a partial small bowel resection was performed for low-flow-associated bowel ischemia. Bowel function recovered. Three weeks after surgery, digital necrosis of both feet was observed clinically. Arterial duplex ultrasound was performed and showed no common femoral artery, profunda femoris artery, superficial femoral artery, or popliteal artery stenosis or occlusion. During recovery, the patient was transferred to a referral hospital. As of June 5, 2020, the patient has not yet been discharged.

Consent has been given by all patients for the publication of the case details and images.

## Discussion

The current outbreak of the SARS-CoV-2 is spreading throughout the globe, causing high morbidity and mortality. Preliminary data have reported an increased risk of venous thromboembolism and acute myocardial infarctions, most likely caused by excessive inflammation, platelet activation, endothelial dysfunction, and stasis.^1^

However, there have also been reports of arterial thrombosis. To investigate the prevalence and incidence of arterial occlusions, we performed a review of the current literature. MEDLINE was searched for peer-reviewed publications on COVID-19 and arterial thromboembolic complications. Four retrospective cohort studies, consisting of a total of 738 patients, and eight case report studies have reported the occurrence of arterial thrombotic events (Table II). In a series of 150 ICU patients referred to four French ICUs reported by Helms et al,^2^ four arterial occlusions were observed, of which one caused mesenteric ischemia, one limb ischemia, and two cerebral ischemia. von Willebrand factor activity, von Willebrand factor antigen, and factor VIII were considerably increased in all patients, and 50 of 57 tested patients (87.7%) had positive lupus anticoagulant.

**Table II:** Studies found in literature review

Study	Study design	No.	Arterial thrombotic events	Type
Helms et al^2^	Multicenter retrospective cohort	150 ICU patients	2 (1.3%) 1 (0.7%) 1 (0.7%)	Ischemic stroke Mesenteric ischemia Limb ischemia
Klok et al^3^	Multicenter retrospective cohort	180 ICU patients	3 (3.7% cumulative incidence)	Ischemic stroke
Lodigiani et al^4^	Single-center retrospective cohort	388 patients	9 (2.5%) 3 ICU, 6 general ward	Ischemic stroke
Bellosta et al^5^	Single-center retrospective cohort	20 patients	20	Acute limb ischemia
Le Berre et al^6^	Case report		1	Intra-aortic thrombus
de Barry et al^7^	Case report		1	Mesenteric thrombosis
Oxley et al^8^	Case report		5	Large-vessel stroke
Vulliamy et al^9^	Case report		1	Aorta-iliac and mesenteric
Avula et al^10^	Case report		4	Ischemic stroke
Giacomelli et al^11^	Case report		1	Aortic prosthetic graft occlusion
González-Pinto et al^12^	Case report		1	Large-vessel stroke
Beyrouti et al^13^	Case report		6	Ischemic stroke

*ICU,* Intensive care unit.

Furthermore, a single-center cohort study from Bellosta et al^5^ reported increased incidence of patients presenting with acute limb ischemia in 2020, 16% vs 2% throughout the same calendar period in 2019. Klok et al^3^ reported that thrombotic complications were observed in 31% of ICU patients in a multicenter cohort of 180 patients admitted to the ICU of three Dutch hospitals. Arterial complications were relatively rare, however, with a cumulative incidence of 3.7%, all of which consisted of ischemic stroke. Similar findings of ischemic stroke occurrence were reported by Lodigiani et al^4^ in a cohort of 388 patients admitted to an academic hospital in Milan, Italy. Nine patients (2.5%) developed ischemic stroke, of whom three patients were at the moment admitted to the ICU and six were on a general ward.

The eight case reports describe the occurrence of acute arterial occlusions in the aorta and mesenteric and cerebral arteries.^6^, ^7^, ^8^, ^9^, ^10^, ^11^, ^12^, ^13^

Complications of COVID-19, including coagulopathy,^14^^,^^15^ may contribute to the development of arterial ischemic events. Elevated D-dimer levels in the setting of COVID-19 have been described in three of the previously described cohort studies.^2^^,^^4^^,^^5^ Furthermore, D-dimer levels were elevated in 15 of 17 patients tested in six identified case reports.^8^, ^9^, ^10^, ^11^, ^12^, ^13^ In our centers, D-dimer levels were determined in one patient and found to be significantly elevated. Although an apparent correlation is suggested, possible causality needs to be investigated. Moreover, COVID-19 causes elevated cytokine levels, including but not limited to tumor necrosis factor α, interleukin (IL) 1β, IL-6, and interferon γ.^16^ Previous research has shown that elevated levels of exogenous tumor necrosis factor α may exacerbate focal ischemic injury in stroke as well as intestinal ischemia in an experimental setting.^17^, ^18^, ^19^ Likewise, IL-1β administration leads to increased infarct size, whereas lack of IL-1β reduces infarct size in experimental focal cerebral ischemia models.^20^ Guidance for considerations in the preventive and therapeutic use of antithrombotic agents as well as potential drug interactions between antiplatelet agents and investigational therapies for COVID-19 has recently been published.^21^ Further research is warranted to elucidate this suggested association between COVID-19 and ischemic complications, its possible underlying pathogenesis, and prevention.

## Conclusions

Our findings demonstrate that physicians should be vigilant for signs of arterial thrombotic complications in COVID-19 patients.
